# The Lipid Profile and Biochemical Parameters of COPD Patients in Relation to Smoking Status

**DOI:** 10.3390/biomedicines10112936

**Published:** 2022-11-15

**Authors:** Cristina Vicol, Ioana Buculei, Oana Elena Melinte, Mona Elisabeta Dobrin, Emanuel Ioan Stavarache, Cristina-Maria Gavrilescu, Paraschiva Postolache, Daniela Matei, Antigona Trofor

**Affiliations:** 1 Department of Biomedical Sciences, Faculty of Medical Bioengineering, University of Medicine and Pharmacy “Grigore T. Popa”, 700115 Iasi, Romania; 2 Doctoral School of the Faculty of Medicine, University of Medicine and Pharmacy “Grigore T. Popa”, 700115 Iasi, Romania; 3 Faculty of Medicine, University of Medicine and Pharmacy “Grigore T. Popa”, 700115 Iasi, Romania; 4 Clinical Hospital of Pulmonary Diseases, 700115 Iasi, Romania; 5 Medical Science Department, University of Medicine and Pharmacy “Iuliu Hațieganu”, 400347 Cluj-Napoca, Romania; 6 Medical Department, Faculty of Medicine, University of Medicine and Pharmacy “Grigore T. Popa”, 700115 Iași, Romania

**Keywords:** tobacco consumption, smoking status, COPD, lipid profile, biochemical parameters, uric acid

## Abstract

Tobacco consumption is the most incriminated and studied risk factor for Chronic obstructive pulmonary disease (COPD), but other factors such as air pollution, are also linked to this disease. One of the known aspects of this chronic lung disease is that its occurrence is mainly due to the chronic inflammation of the airways. Lipid metabolism seems to be affected by smoking, with studies showing a correlation between this habit and high levels of triglycerides and low levels of high-density lipoprotein cholesterol (HDL-CHOL). Uric acid concentration is thought to reflect the antioxidative capacity of the body because it is the most abundant aqueous antioxidant. The aim of this study was to investigate the lipid profile and biochemical parameters of COPD patients in relation to smoking status. The present study was conducted between 2020 and 2021 in the Clinical Hospital of Pneumology in Iasi, Romania. Patients diagnosed with COPD (n = 52) were included and divided in three groups depending on their smoking status: non-smokers, smokers and ex-smokers. The obtained results show low correlations between COPD stages and serum uric acid concentrations (r = 0.4; *p* ˂ 0.05), smoking status (smoker/non-smoker/ex-smoker) and total serum cholesterol values (r = 0.45; *p* ˂ 0.05), but also between serum urea concentrations and the number of packs-years for the smoker/ex-smoker groups (r = 0.45, *p* ˂ 0.05). Smoking was associated with changes in the lipid profile of smokers and ex-smokers, along with increased low-density lipoprotein cholesterol (LDL-CHOL) and low serum uric acid values.

## 1. Introduction

Chronic obstructive pulmonary disease (COPD) is defined as a preventable disease, caused mainly by chronic inflammation of the airways, characterized by persistent symptoms such as cough and dyspnea, and modification of lung function parameters shown on spirometry. Smoking is the most incriminated and studied risk factor for COPD, but other factors such as air pollution, are also linked to this disease. The World Health Organization (WHO) states that over 400 million people suffer from COPD and that this disease is the third highest cause of mortality worldwide [1].

One of the known aspects of this chronic lung disease is that its occurrence is mainly due to the chronic inflammation of the airways. Tobacco smoke causes oxidative stress which plays an important role in the onset and maintenance of inflammation in the respiratory tract. The acceleration of the inflammatory reaction is due to inflammatory cytokines such as TNF-α, TNF-β and IL-6 in murine alveolar epithelial type II cells and IL-8 in the airway cells generated due to the oxidative stress favoring an increased expression of these inflammatory cytokines by messenger RNA which facilitates NK-kB binding to DNA [2]. Another determinant of the development, but especially the progression of COPD, is systemic inflammation. Exposure to tobacco smoke can lead to its occurrence and maintenance either by direct or indirect mechanisms [3].

Chronic tobacco use is defined as tobacco or nicotine dependence. Assessment of nicotine dependence may be performed clinically or paraclinically. The clinical assessment is based on the determination of smoking status, type of tobacco product used, tobacco use and nicotine dependence. By investigating the number of cigarettes smoked, we can determine the status of smoking, respectively: non-smoker, occasional smoker, regular smoker or former smoker. Tobacco use can be defined as the number of cigarettes smoked in a day or the number of packs of cigarettes/years (PY). Nicotine dependence can be diagnosed using the Fagerström nicotine dependence test. Paraclinical assessment of tobacco dependence can be performed by laboratory biochemical tests, which highlight the presence of biomarkers of exposure to tobacco smoke. These tests include the concentration of carbon monoxide in the exhaled air and the level of cotinine, a metabolite of nicotine, which can be identified in urine, serum, saliva, etc. The most commonly used biomarkers in research to confirm or deny self-reported abstinence are carbon monoxide in exhaled air, cotinine (a metabolite of nicotine, measurable in plasma, saliva, urine, hair and intranasally), but also markers such as anatabine, anabazine, thiocyanate, uric acid (UA) and nitric oxide [4]. Another biomarker of exposure to tobacco smoke is uric acid, the degradation product of nucleic acids and the final product of purine oxidation. It is transported by plasma from the liver to the kidneys where it is filtered and then excreted in a percentage of 70%, the rest being degraded in the gastrointestinal tract. Uric acid may vary depending on age, diet, sex, genetic factors, exercise, menopause, etc. [5]. UA concentration is thought to reflect the antioxidative capacity of the body because it is the most abundant aqueous antioxidant. Smoking causes oxidative stress and uric acid is responsible for up to 60% of serum free radical scavenging [6].

Lipid metabolism seems to be affected by smoking, with studies showing a correlation between this habit and high levels of triglycerides and low levels of high-density lipoprotein cholesterol (HDL-CHOL) [7]. The mechanism by which smoking alters the lipid profile is still not fully understood, but several hypotheses have been proposed in the specialized literature. One of these hypotheses states that nicotine stimulates the production and secretion of growth hormones, catecholamines and cortisol, which causes an increase in the serum concentrations of free fatty acids, which triggers the hepatic secretion of triglycerides and very-low-density lipoprotein (VLDL-C) [8]. In one study, Khojah and Ahmed found that smokers compared to non-smokers have higher levels of triglycerides, cholesterol, VLDL-C, low-density lipoprotein cholesterol (LDL-CHOL) and lower levels of high-density lipoprotein cholesterol (HDL-CHOL) [9]. The effects of chronic tobacco consumption (smoking and chewing) on the lipid profile of 75 individuals divided in three groups (I: non-smokers and non- chewers; II: smokers and non-chewers and III: chewers and non-smokers) were studied by Rao and Subash and the results showed that the levels of cholesterol, VLDL-C, LDL-CHOL and triglycerides were higher in group II and III compared to group I, and on the other hand, HDL-CHOL had decreased levels in the same groups of patients [10]. These findings seem to reflect the impact that smoking has on lipid metabolism. In smokers, a lower activity of lecithin cholesterol acyltransferase (LCAT) and lipoprotein lipase (LPL) and a higher activity of cholesterol ester transfer protein (CETP) was observed [11,12,13].

The aim of this study was to investigate the lipid profile and biochemical parameters of COPD patients in relation to smoking status.

## 2. Materials and Methods

### 2.1. Study Design and Setting

The present study was conducted between 2020–2021 in the Clinical Hospital of Pneumology in Iasi, Romania. This hospital is a reference center for the treatment of pulmonary diseases in the north-east region of Romania.

### 2.2. Patient Selection

Patients diagnosed with COPD (n = 52) were included and voluntarily participated in this study. An informed consent was obtained from every participant in the study. The study was approved by the Ethics Committee of Iasi Clinical Hospital of Pneumology (Iasi, Romania) and by the Ethics Committee of the University of Medicine and Pharmacy, Grigore T. Popa” (Iasi, Romania).

The present study included patients who were able to understand and comply with the study procedures, who signed the informed consent and were diagnosed with COPD according to the GOLD criteria. Patients who did not corresponded to the above-mentioned criteria, patients who did not agree to participate in the study by signing the informed consent, who were not able to understand the study protocol, who suffered from unstable acute or chronic medical conditions (cardiovascular diseases, psychiatric diseases, etc.), who had neuro-motor retardation, pregnant women and patients who were under treatment with statins, allopurinol and/or colchicine were excluded.

Patients were recruited at the Clinical Hospital of Pneumology in Iasi, between 2020 and 2021, and the diagnosis of COPD was obtained using anamnestic and clinical data, medical documents, current symptoms and was confirmed using lung function tests, respectively of the value of spirometric ratio forced expiratory volume in one second (FEV1)/forced vital capacity (FVC) < 0.70, according to the Global Initiative for Chronic Obstructive Lung Disease (GOLD) recommendations [14]. Patients were subdivided by the severity of airflow limitation assessed by FEV1 as follows: FEV1 value below 80% equals GOLD I stage, FEV1 value between 50 and 80% represent GOLD II stage, FEV1 value between 30 and 50% represent GOLD III stage, and FEV1 value below 30% equals GOLD IV stage.

All the subjects included in the study received treatment for COPD with B2 agonists in association with other bronchodilators and/or inhaled corticosteroids.

A division of the patients in different groups depending on the status of smoking was performed: patients who smoked on a daily basis were included in the smoker group, patients who smoked less than 100 cigars all their life were included in the non-smoker group and patients who quit smoking more than 6 months before examination were included in the ex-smoker group.

### 2.3. Data Collection and Management

Electronic medical records were retrospectively screened and relevant data were independently extracted for all the participants included in the study. The relevant data extracted were included in a de-identified form into an excel spreadsheet. All data was stored in password protected electronic documents with access only for the authors of the study.

Patient demographic data and baseline clinical characteristics including age, gender and comorbidities were recorded. Clinical data consisted of symptoms and smoking status. Paraclinical data consisted of lung function test results, lipid profile and biochemical parameter values.

### 2.4. Biochemical Assay

Serum parameters were measured using a Cobas Integra 400 plus (Roche) biochemical auto analyzer. All tests were performed at the Biochemical Laboratory, following standard procedures for clinical biochemistry purposes. The biological markers measured were urea, creatinine, glucose, total cholesterol, triglycerides, HDL-CHOL, LDL-CHOL and uric acid. Total lipids values were calculated using formulas based on enzymatic measurements of triglycerides and total cholesterol [TL = 1.33 ⁎ TG + 1.12 ⁎ CHOL + 1.48(g/l)] [15].

### 2.5. Statistical Analysis

This study evaluated the clinical features and biological variables of 52 patients diagnosed with different stages of COPD. The statistical analysis of the data was performed using the Statistics 10 program and highlighted the statistical results of the patients investigated at the Clinical Hospital of Pneumology, Iasi. Distribution of the data was evaluated with Kolmogorov–Smirnov test, a *p*-value < 0.05 was considered to indicate statistical significance and distribution of the data regarding the lipidic profile were evaluated using Q–Q plot diagrams. Mann–Whitney U test was used to compare the biochemical parameters for two independent groups (smokers vs. non-smokers, smokers vs. ex-smokers, non-smokers vs. ex-smoker). Descriptive statistics in case of smokers/non-smokers/ex-smokers were present in terms of average, standard deviation, median and range. Spearman’s rank correlation coefficient was used to measure the strength of the correlations between biochemical parameters corresponding to smokers and non-smokers.

## 3. Results

In this observational study, 52 patients suffering from COPD were included; clinical features and biological variables were statistically assessed. Table 1 shows the characteristics of the patients included in the study and Table 2 shows the descriptive statistics of the biochemical parameters of the patients included in the study. The median age of the actively smoking patient was 65 years old. A predominance of the male gender was observed in all groups; in the smoker’s group, 88% of the participants were male (Table 1).

**Figure 1:**
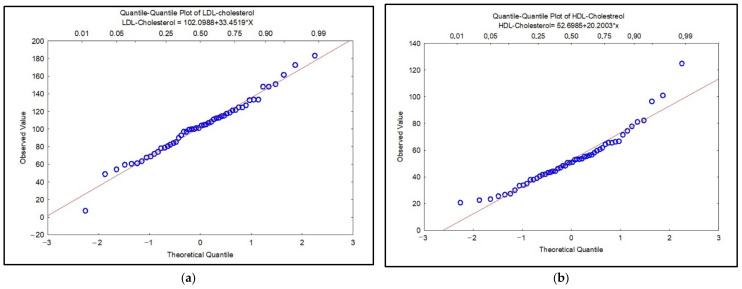

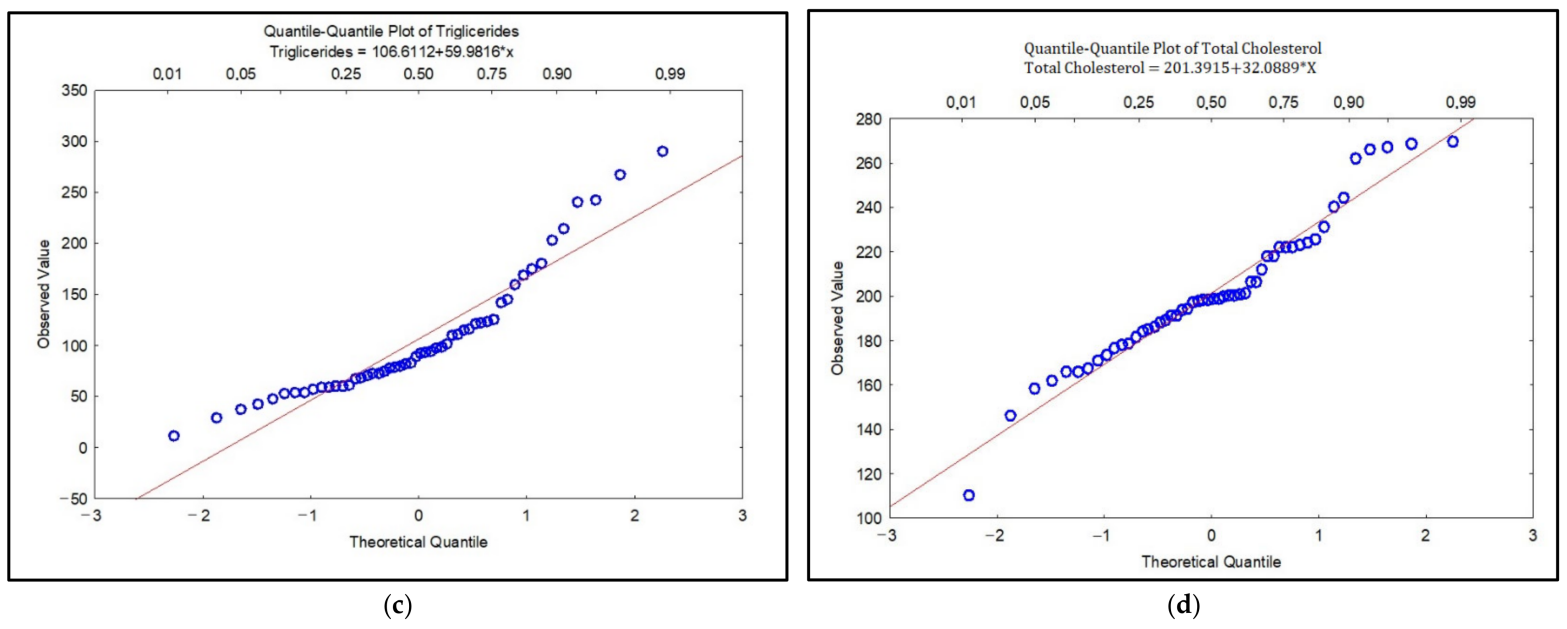
Q–Q plot for LDL-cholesterol (mg/dL) (**a**); HDL-cholesterol (mg/dL) (**b**); triglycerides (**c**) and total cholesterol (**d**).

Regarding the stage of the disease, smokers in the investigated group were categorized as COPD Stage I: 12%, COPD Stage II: 19%, COPD Stage III: 29% and COPD Stage IV: 40%; non-smokers were categorized as COPD Stage III: 62%, Stage IV: 38%; and former smokers were categorized as COPD Stage II: 26%, COPD Stage III: 42% and COPD Stage IV: 32% (Table 1). The results of the statistical analysis in the case of nonparametric tests showed that the investigated data are not normally distributed (*p* ˂ 0.05) and the Spearman correlation coefficients revealed statistical significance between the investigated clinical parameters with values for r = 0.28–0.94, *p* ˂ 0.05. Figure 1 shows the distribution of data by Quantile–Quantile plot (Q–Q plot) diagrams for total cholesterol, HDL-CHOL, LDL-CHOL and triglycerides in COPD patients. In Figure 1a–c, the visually check can be observed for the fit of a theoretical distribution to the observed data by examining the quantile–quantile (or Q–Q) plot. In this plot, the observed values of a variable are plotted against the theoretical quantiles. A good fit of the theoretical distribution to the observed values would be indicated by this plot if the plotted values fall onto a straight line. We also observed that the data are not normally distributed in Figure 1c, d because the results exceed the straight line. The resulting plot is a scatterplot of the observed values against the expected values, given the respective distribution.

**Figure 2:**
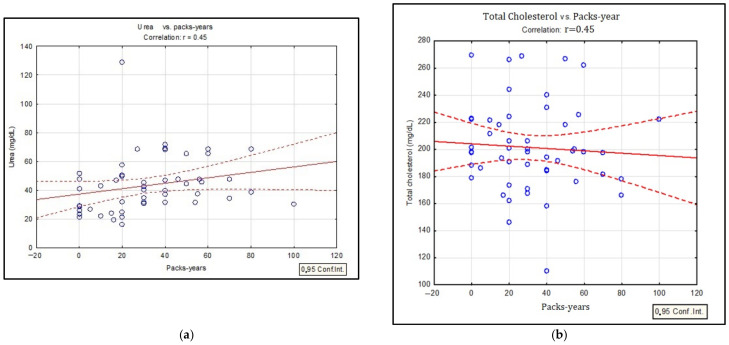

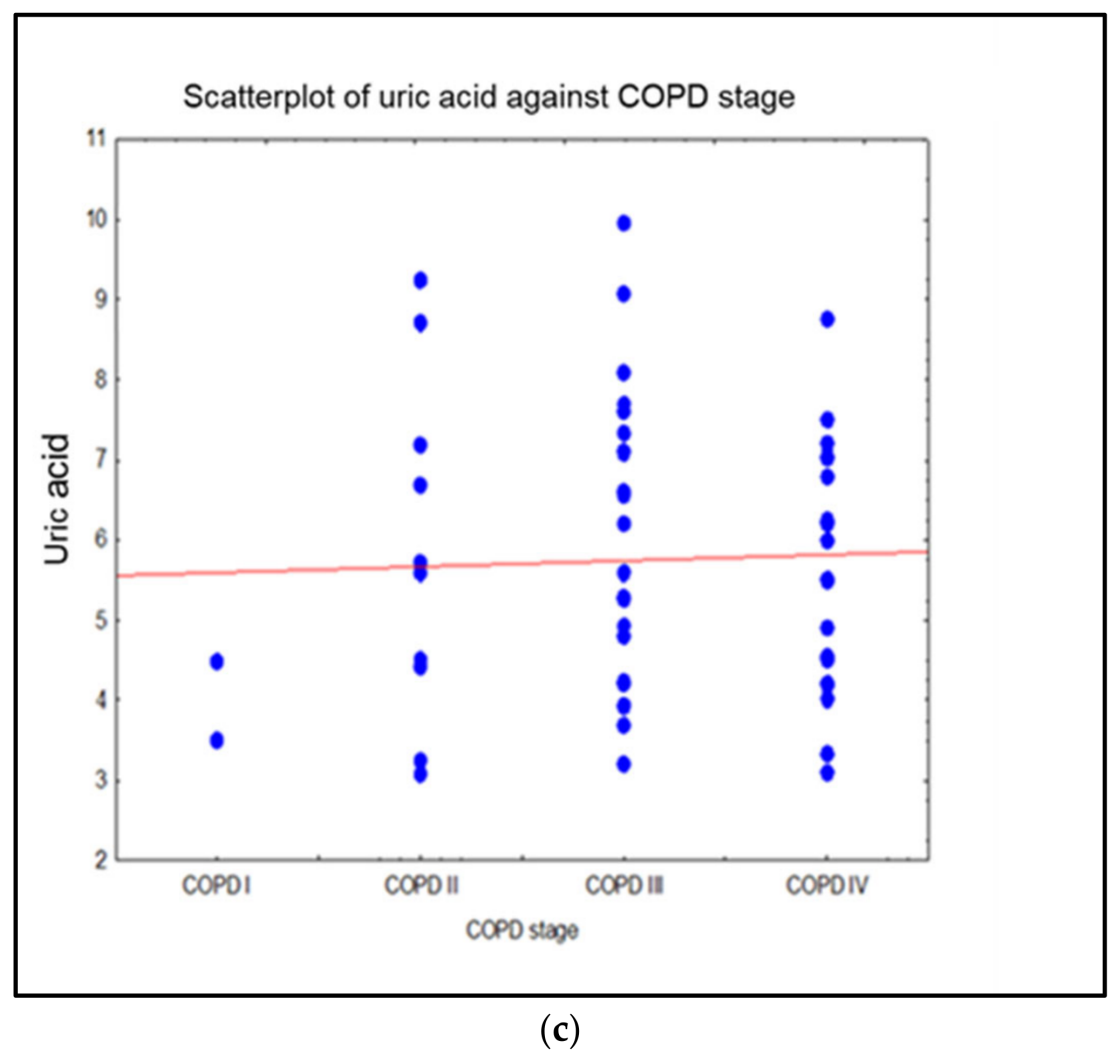
Linear regression between urea and PY (packs-years) (**a**); cholesterol and PY (packs-years) (**b**); uric acid and COPD stage (**c**).

The statistical difference between the investigated smoker/non-smoker/ex-smoker groups was assessed with the Mann–Whitney U test. Thus, the results showed a statistically significant difference between the smoker/ex-smoker groups given by the following biological parameters: total cholesterol (*p* = 0.012), LDL-CHOL (*p* = 0.039), PY (*p* = 0.006). The results from the descriptive statistics point to LDL-CHOL values for smokers/ex-smokers 106.34 versus 110.53 mg/dL, slightly higher than the reference biological range compared to the mean LDL-CHOL concentration values in non-smokers (98.28 mg/dL). Low correlations were obtained between COPD stages and serum uric acid concentrations (r = 0.4; *p* ˂ 0.05), smoking status (smoker/non-smoker/ex-smoker) and total serum cholesterol values (r = 0.45; *p* ˂ 0.05), but also between serum urea concentrations and the number of packs-years for the smoker/ex-smoker groups (r = 0.45, *p* ˂ 0.05) (Figure 2).

**Figure 3:**
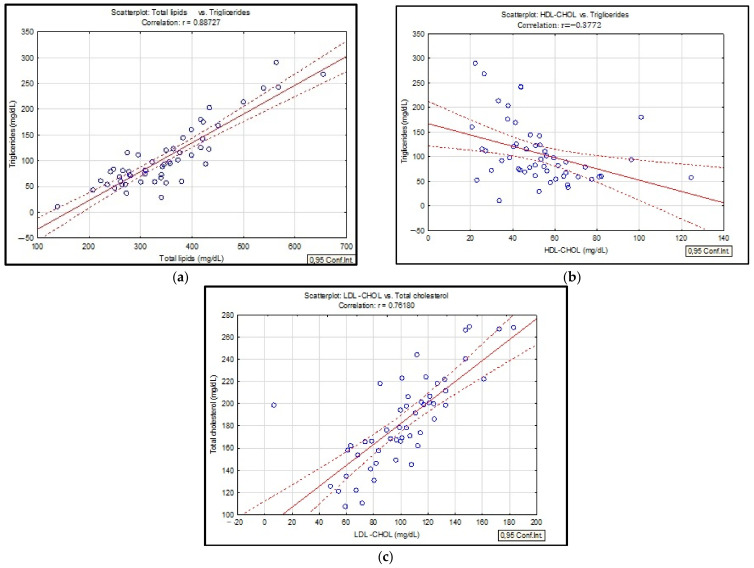
Linear regression between total lipids and triglycerides (**a**); triglycerides and HDL-CHOL (**b**); LDL-CHOL and total cholesterol (**c**) in patients with COPD.

Analyzing the correlation matrix (Table 3) for the three investigated groups of smokers/non-smokers/ex-smokers, statistically significant Spearman correlation coefficients were obtained. Thus, evaluating the lipid profile, significant statistical correlations were observed between total lipid values and triglyceride values (r = 0.88, *p* ˂ 0.05), LDL-CHOL concentrations and serum total cholesterol (r = 0.76, *p* ˂ 0.05) and also between the values of serum triglycerides and HDL-CHOL (r = 0.37, *p* ˂ 0.05) (Figure 3).

**Figure 4:**
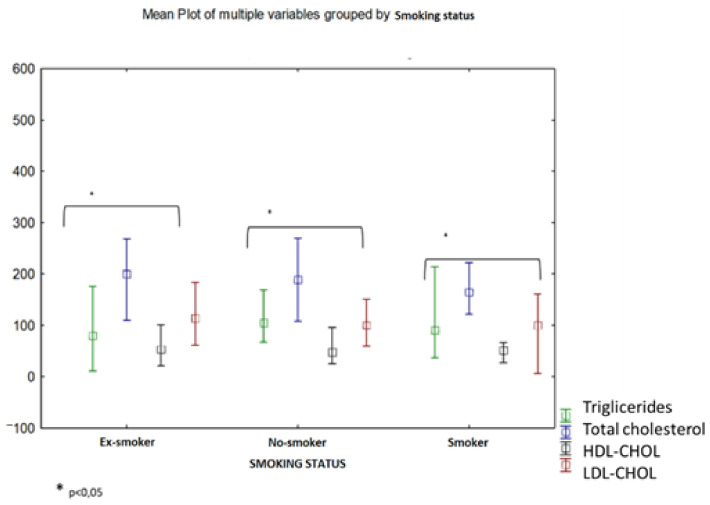
Lipidic profile (triglycerides, total cholesterol, HDL-CHOL, LDL-CHOL) in blood serum of COPD patients depending on the smoking status (smokers/non-smokers/ex-smokers).

Moreover, the variations of serum lipids in case of the three investigated groups were characterized by increased levels of serum triglycerides, low values of serum HDL-CHOL as well as an increased level of LDL-CHOL. The results of the present study point to low values of HDL-CHOL in smokers (mean concentration = 45.82 mg/dL) but also for ex-smokers (mean concentration = 52.48 mg/dL) compared to non-smoking patients (mean concentration = 53.15 mg/dL) (Figure 4).

In Table 4, the lipid profile of the investigated patients was evaluated depending also on the demographic area and comorbidities shown in Table 1. The statistical results evaluated with the Mann–Whitney U test for urban/rural area differentiation pointed out that there are no statistically significant differences (*p* ˃ 0.05) in the assessment of the demographic area of the patients included in the present study. The level of serum lipids was evaluated according to comorbidities; the statistical results showed that there were no statistically significant differences, with the exception of obesity (*p* = 0.01).

## 4. Discussion

In this study, the associations between smoking status and biochemical indicators in case of patients diagnosed with COPD were investigated. The present study correlates tobacco use and COPD with lipidic profile markers useful in clinical practice. Antioxidant status was evaluated according to the level of uric acid, smoking was quantified using packs-years (PY) and other useful parameters were also assessed: glucose, urea and creatinine. The results obtained in the present study point to a low level of uric acid in the case of smoking patients and positive correlation with COPD stages. Studies in this field have shown that UA levels are higher in COPD patients compared to healthy people, suggesting that tissue hypoxia causes an increase in purine catabolism, especially in the advanced stages of the disease [16]. Mouhamed et al. conducted a study about the effects of smoking on uric acid concentrations showing that plasma uric acid concentrations were lower in smokers versus non-smokers. The author attributed this finding to the fact that smoking reduces the endogenous production because of the oxidative stress generated. In fact, serum uric acid acts as a true antioxidant, including against oxidative stress induced by tobacco use. Thus, low serum uric acid concentrations in smokers have been attributed to decreased endogenous production as a result of chronic exposure to tobacco smoke, which is a significant source of oxidative stress. Increased oxidative stress can lead to depletion of antioxidants, including uric acid [6]. In fact, cigarette smoke, due to the large number of chemical substances resulting from combustion (4700 substances, including polycyclic aromatic hydrocarbons, heavy metals, NO) leads to the formation of a strong chemical oxidative stress involving practically all the compounds present in tobacco, leading to the formation of free radicals. In this case, free radicals are able to react with all the constituents of the cell and the matrix, resulting in the so-called state of oxidative stress [17]. Another study showing the same results was conducted by Hanna et al., where correlations between smoking status and uric acid concentrations were also observed [18]. Similar results of serum uric acid concentrations for smokers/non-smokers were presented in recent studies [19], and low values were associated with oxidative stress that reduces antioxidant levels.

Recent studies regarding imbalances in lipid metabolism point to the fact that smoking as well as oxidative stress are possible mechanisms responsible for the development of dyslipidemia in COPD patients. However, there are limitations to these statements that are related to the severity of the disease, gender, body mass index (BMI) and especially to the intensity of smoking [20,21]. Furthermore, literature point that smoking is associated with an atherogenic lipid profile, which can also contribute to the production of oxidative stress. Smoking, in its various forms, leads to an increased risk for high total cholesterol serum levels, as well as for high triglycerides levels [19]. In this study, the parameters of the lipidic profile were observed to not be normally distributed (Figure 1a–d) and the statistical results presented in Figure 2 show important correlations especially for total cholesterol, triglycerides, total lipids and LDL-CHOL.

Several hypotheses about the way in which smoking modifies the values of serum lipids were proposed. It is assumed that an increase in the level of catecholamines can lead to an increase in circulating free fatty acids with a role in modifying lipid metabolism, which also stimulates the hepatic production of triglycerides [22]. In this study, we also observed high values of triglycerides in smokers and important correlation with total lipids (Figure 2). Literature studies associate increased serum concentrations of triglycerides with decreased lipoprotein lipase activity in smokers, an enzyme of particular importance in the synthesis of triglycerides [23]. Currently, there are several explanations regarding the changes in the lipid profile of COPD patients. An important factor is systemic inflammation associated with both increased serum triglycerides and decreased HDL-CHOL. The same associations were observed in the present study: decreased HDL-CHOL in smokers and high levels for LDL-CHOL (Table 2). The most reasonable explanation for the pathological values of HDL-CHOL would be the administration of drugs based on β-2 agonists which are recommended for the treatment of COPD [24,25,26]. In the case of the present study, no significant correlations were found between the administration of β-2 agonists and HDL-CHOL values (Table 3). At the same time, a study conducted on patients with COPD showed that the administration of corticosteroids, especially in patients with exacerbations, significantly affects the level of plasma lipids [27]. Moreover, additional studies should be carried out to evaluate the change in the level of serum lipids following the administration of corticosteroid treatment. In this hypothesis, it is assumed that pro-inflammatory cytokines would produce an imbalance in lipid metabolism, which can be quantified by low levels of HDL-CHOL and increased concentrations for IL-6 [28]. Another explanation for the change in the lipid profile of COPD patients is the fact that these types of patients are physically inactive in their daily life, especially those diagnosed with Stage IV, which could increase their risk of dyslipidemia [29]. Studies have shown that in patients diagnosed with COPD the prevalence of metabolic syndrome is between 20–50% [29,30]. Śliwińska-Mossoń et al. showed in a study that LDL-CHOL concentrations were 1.5 higher in smokers compared to non-smokers [31]. In a study conducted by Szkup M. et al. on women smokers, the same results were obtained [32]. Dyslipidemia has also been observed to be a major risk factor for metabolic syndrome and cardiovascular disease and is accompanied by variations in serum lipids.

There are various factors that can modify the lipid profile in humans, such as exposure to tobacco smoke and exposure to air pollution. Results of a study conducted in this field provide strong evidence that long-term exposure to air pollution affects lipid profiles in humans. These findings shed new light on the importance of controlling air pollution to prevent changes that lead to abnormal blood lipid profiles, which are an independent risk factor for cardiovascular diseases and other diseases [33]. Another study showed the same results and concluded that long-term ambient air pollution was associated with both altered lipid profiles and dyslipidemia, particularly among overweight or obese participants [34]. The same results were obtained in the present study, regarding obesity and total lipids (Table 4). Smoking has been shown to be an independent risk factor for diabetes, and together with genetic factors and obesity, is one of the main risk factors for insulin resistance [35,36]. At the same time, some studies have pointed out the presence of oxidative stress in T2D (Type 2 diabetes) due to the activation of a specific biochemical pathway with a role in increasing the production of reactive oxygen species. In smoking patients with COPD, oxidative stress induced by tobacco smoke could influence insulin resistance [37]. Moreover, in patients with COPD, short-term treatment with oral corticosteroids was associated with a five times higher risk of acute hyperglycemia. Long-term treatment with oral corticosteroids has been associated with an increased risk of glucose intolerance [38].

The relationship between smoking and serum creatinine levels has been discussed in few studies. What was observed is that the level of serum creatinine increases in active smokers [39], as well as in the case of subjects with various renal diseases and hypertension [40]. Dülger et al. studied the renal function in active and passive smokers and showed that creatinine levels were significantly increased in active smokers (*p* < 0.01) and concluded that kidneys, and in particular the glomerular function, can be affected even in case of passive smoking [39]. Few studies in the literature showed that smoking increases the risk of proteinuria and also the risk of mild hyperfiltration, as well as the risk of mild renal impairment, especially in men and in elderly people. Smoking in general has a negative influence on renal function even in subjects apparently not suffering from a kidney disease, but the adverse renal effects due to smoking are present especially in patients with different kidney disorders as well as in hypertensive patients [19].

## 5. Conclusions

COPD is considered a systemic disease, and tobacco consumption is an important contributor to the development of this pathology. Tobacco smoking was also associated with dyslipidemia. In the case of the patients included in the present study, smoking was associated with changes in the lipid profile of smokers and ex-smokers and these results were highlighted by increased serum triglyceride concentrations, decreased plasma high density lipoproteins and HDL-CHOL and increased LDL-CHOL fraction. Low serum uric acid values in smokers could identify a chemical oxidative stress produced by the effect of toxic compounds in tobacco smoke, which in turn could trigger an imbalance of lipid metabolism in smokers. More studies need to be conducted in this field to obtain a better understanding of the mechanisms involved in the alteration of the lipid profile of smoker and ex-smoker patients.

## Figures and Tables

**Table 1:** Characteristics of the patients included in the study.

Characteristic	Smokers	Non-Smokers	Ex-Smokers	*p*-Value
(N = 52)	N = 16	N = 8	N = 28	
Median (± SDEV)-age-years	65 ± 8.82	69 ± 9.35	68 ± 8.91	
Male N/%	14/88	7/88	26/93	0.07
Female N/%	2/12	1/12	2/7	0.69
Urban/Rural area %/%	50/50	33/67	46/54	0.46
COPD I N/%	2/12	-	-	0.06
COPD II N/%	3/19	-	7/26	0.05
COPD III N/%	5/29	5/62	12/42	
COPD IV N/%	6/40	3/38	9/32	
The “ABCD” assessment tool				
A N/%	0	0	1/4	
B N/%	4/25	3/38	10/36	
C N/%	0	0	2/7	
D N/%	12/75	5/63	15/54	
FEV1 (mean/median) %/%	37.10/34.55	42.02/37.35	48.53/43.80	
PY (mean/median)	50.81/43.0	-	30.18/28.5	
Comorbidities				
Hypertension N/%	5/56	5/62	15/56	
Diabetes mellitus N/%	3/19	1/13	3/11	
Obesity N/%	1/6	2/25	4/14	

COPD I—Chronic obstructive pulmonary disease Stage I; COPD II—Chronic obstructive pulmonary disease Stage II; COPD III—Chronic obstructive pulmonary disease Stage III; COPD IV—Chronic obstructive pulmonary disease Stage IV; FEV1—forced expiratory volume in one second; PY—(number of packs of cigarettes/years).

**Table 2:** Descriptive statistics of the biochemical parameters of the patients included in the study (all parameters are expressed in mg/dL).

Parameters *	Smokers				Non-Smokers				Ex-Smokers			
COPD Patients	Average	SD	Median	Range	Average	SD	Median	Range	Average	SD	Median	Range
	(N = 16)				(N = 8)				(N = 28)			
Uric acid	5.28	1.80	5. 22	3.21–9.08	5.93	1.77	5.42	3.10–7.70	5.86	1.78	5.60	3.09–9.97
Urea	49.23	14.54	47.60	30.40–68.9	33.66	11.49	29.10	21.50–51.60	42.97	22.39	40.10	16.20–129
Creatinine	0.81	0.23	0.78	0.50–1.16	0.74	0.28	0.70	0.35–1.23	0.79	82.07	0.72	0.35–2.40
Glucose	104.26	20.16	96.00	83.20–140.40	105.90	30.33	109.60	77.70–134.00	109.34	24.09	106.40	68.80–162.50
Triglycerides	110.32	62.10	90.26	36.82–240.81	105.03	32.37	104.25	67.00–168.50	104.95	69.03	83.82	10.76–290.00
Total Cholesterol	200.2	29.03	194.05	121.11–222.07	183.49	52.55	188.52	107.30–269.40	196.34	39.83	199.34	110.20–268.60
Total lipides	370.98	98.74	288.57	207.93–538.9	346.68	71.54	345.37	241.71–450.82	360.96	107.60	348.41	139.21–655.42
HDL-CHOL *	45.82	11.49	50.61	27.18–66.36	53.15	21.37	47.30	25.58–96.19	52.48	24.76	52.60	20.73–124.64
LDL-CHOL **	106.34	41.29	99.89	6.84–161.14	98.26	47.57	100.045	59.13–150.73	110.53	30.47	113.34	61.17–183.26

* HDL-CHOL—High density lipoproteins-cholesterol; ** LDL-CHOL—Low density lipoproteins cholesterol.

**Table 3:** Application of the Spearman correlation to biochemical parameters and long-acting beta 2-agonists in case of patients with COPD.

	UA	Urea	Creatinine	Glucose	Triglicerides	CHOL	LipT	HDL-CHOL	LDL-CHOL	COPD Stage	Status of Smoking	PY	VEMS	LABA
UA	1													
Urea	0.281	1												
Creatinine	0.546	0.500	1											
Glucose	0.316	0.007	0.456	1										
Triglicerides	0.125	0.357	0.037	0.004	1									
CHOL	0.048	0.105	0.014	0.044	0.089	1								
LipT	0.031	0.308	0.046	0.060	0.884	0.945	1							
HDL-CHOL	0.007	0.144	0.113	0.006	0.377	0.220	0.234	1						
LDL -CHOL	0.078	0.154	0.018	0.089	0.209	0.809	0.945	0.043	1					
COPD stage	0.398	0.028	0.231	0.273	0.057	0.056	0.061	0.046	0.059	1				
Status of smoking	0.025	0.182	0.144	0.067	0.140	0.453	0.120	0.09	0.297	0.204	1			
PY	0.238	0.451	0.139	0.170	0.046	0.134	0.030	0.069	0.141	0.211	0.215	1		
VEMS	0.180	0.044	0.072	0.039	0.227	0.117	0.100	0.188	0.068	0.142	0.252	0.068	1	
LABA	0.188	0.073	0.086	0.191	0.138	0.124	0.105	0.093	0.134	0.070	0.042	0.180	0.047	1

* LABA–Long-acting beta2-agonists.

**Table 4:** Mann–Whitney U test applied for comorbidities and demographic area of COPD patients.

	Mann-Whitney U Test by Variable Obesity; Marked Tests are Significant at *p* < 0.05000									
	Rank Sum	Rank Sum	U	Z	*p*-Value	Z	*p*-Value	Valid N	Valid N	2*1sided
Total cholesterol	1224.50	153.50	125.50	0.84	0.40	0.84	0.40	45.00	7.00	0.40
LipT	1287.00	91.00	63.00	2.52	0.01	2.52	0.01	45.00	7.00	0.01
HDL-CHOL	1245.00	133.00	105.00	1.39	0.16	1.39	0.16	45.00	7.00	0.17
LDL -CHOL	1211.00	167.00	139.00	0.48	0.63	0.48	0.63	45.00	7.00	0.64
Triglicerides	1217.00	161.00	133.00	0.64	0.52	0.64	0.52	45.00	7.00	0.53
	**Mann-Whitney U Test by variable Diabetes mellitus; Marked tests are significant at *p* < 0.05000**									
	Rank Sum	Rank Sum	U	Z	*p*-value	Z	*p*-value	Valid N	Valid N	2*1sided
Total cholesterol	1152.00	226.00	117.00	−1.07	0.28	−1.07	0.28	45.00	7.00	0.29
LipT	1157.00	221.00	122.00	−0.94	0.35	−0.94	0.35	45.00	7.00	0.36
HDL-CHOL	1209.00	169.00	141.00	0.43	0.67	0.43	0.67	45.00	7.00	0.67
LDL -CHOL	1207.00	171.00	143.00	0.38	0.71	0.38	0.71	45.00	7.00	0.71
Triglicerides	1252.00	126.00	98.00	1.58	0.11	1.58	0.11	45.00	7.00	0.12
	**Mann-Whitney U Test by variable hypertension; Marked tests are significant at *p* < 0.05000**									
	Rank Sum	Rank Sum	U	Z	*p*-value	Z	*p*-value	Valid N	Valid N	2*1sided
Total cholesterol	580.00	798.00	304.00	−0.53	0.59	-0.53	0.59	23.00	29.00	0.60
LipT	527.00	851.00	251.00	−1.51	0.13	−1.51	0.13	23.00	29.00	0.13
HDL-CHOL	558.00	820.00	282.00	−0.94	0.35	−0.94	0.35	23.00	29.00	0.35
LDL-CHOL	526.50	851.50	250.50	−1.52	0.13	−1.52	0.13	23.00	29.00	0.13
Triglicerides	607.50	770.50	331.50	−0.03	0.98	−0.03	0.98	23.00	29.00	0.97
	**Mann-Whitney U Test by variable urban/rural area; Marked tests are significant at *p* < 0.05000**									
	Rank Sum	Rank Sum	U	Z	*p*-value	Z	*p*-value	Valid N	Valid N	2*1sided
Triglicerides	780.50	597.50	246.50	1.67	0.10	1.67	0.10	26.00	26.00	0.09
Total cholesterol	718.00	660.00	309.00	0.52	0.60	0.52	0.60	26.00	26.00	0.60
HDL-CHOL	663.00	715.00	312.00	−0.47	0.64	−0.47	0.64	26.00	26.00	0.64
LDL-CHOL	786.00	592.00	241.00	1.77	0.08	1.77	0.08	26.00	26.00	0.08
LipT	607.50	670.50	331.50	−0.03	0.98	−0.03	0.98	26.00	26.00	0.97

## Data Availability

Not applicable.
